# An easy-to-use laryngostroboscopy index for unilateral vocal fold paralysis reflects voice quality

**DOI:** 10.1007/s00405-026-10575-4

**Published:** 2026-09-16

**Authors:** Alexander Mainka, Dirk Mürbe, Mario Fleischer, Tadeus Nawka

**Affiliations:** Department of Audiology and Phoniatrics, Charité - Universitätsmedizin Berlin, Corporate Member of Freie Universität Berlin and Humboldt-Universität zu Berlin, Charitéplatz 1, Berlin, 10117 Berlin Germany

**Keywords:** Laryngostroboscopy evaluation protocol, Vocal fold paralysis, Mucosal wave, Glottal gap, Numerical analysis, Validation

## Abstract

**Background:**

To date, laryngostroboscopic parameters have been rarely used as outcome measures in clinical interventional studies, due tomethodological difficulties.

**Methods:**

We applied a greatly reduced laryngostroboscopy evaluation protocol to an intervention study for unilateral vocal fold paralysis. Here,we investigate the correlation between the newly designed laryngostroboscopy index for unilateral vocal fold paralysis (LSI-UVFP) andan expert group’s rating of voice quality. The LSI UVFP evaluates four key parameters closely related to voice production in UVFPpatients: (1) the vocal fold position, (2) the glottal gap during the closed phase, (3) atrophy of the paralysed vocal fold, and (4) themucosal wave. Each parameter is classified on a four-point ordinal scale: 0 for normal, 1 for mild, 2 for moderate, and 3 for severe. 52video recordings from 26 patients, before and after a voice improving surgery, were evaluated using the LSI-UVFP.

**Results:**

The index demonstrates good reliability, with an intra-class correlation coefficient of 0.80. In addition, LSI-UVFP was found to show asignificant correlation with an auditory perceptual rating of voice quality.

**Conclusion:**

Thus, rating of laryngostroboscopy by the LSI-UVFP is a valid method for documenting and comparing therapeutic outcomes ininterventional studies for symptomatic UVFP. Its usage enables a meaningful interpretation of videolaryngostroboscopic data withregard to voice quality.

**Supplementary Information:**

The online version contains supplementary material available at https://doi.org/10.1007/s00405-026-10575-4.

## Introduction

Laryngostroboscopy is a standard diagnostic tool for voice disorders. It is imperative for an accurate assessment of voice disorders. Medical societies, such as the Union of European Phoniatricians and the European Laryngological Society, have long recommended its use as the primary clinical tool for the aetiological diagnosis of laryngeal diseases. In a consensus paper, they state that the symmetry, amplitude, closure, regularity, movements, and morphology of the results of laryngostroboscopy allow a detailed description of the vibratory process of the vocal folds  [1].

Several authors have published proposals for quantitative assessment. Poburka developed a stroboscopy rating form in 1999 [2]. Rosen studied stroboscopy as a research instrument [3]. Uloza proposed a video laryngostroboscopy assessment form with glottic closure, regularity, mucosal wave, glottal image symmetry, and vibration symmetry as parameters [4]. Ricci-Maccarini just recently published data on another laryngostroboscopy protocol [5]. However, in a current review on laryngeal reinnervation, glottic closure, and vocal fold motion were the only examinable parameters extracted from laryngostroboscopy [6].

The use of a quantitative assessment is desirable to utilise laryngostroboscopic findings not only for diagnostic purposes, but also to evaluate treatment outcomes. Given the plethora of laryngostroboscopic evaluation protocols, it is necessary to define the outcome parameters in a standardised evaluation protocol. In a systematic review of the parameters and scales used to assess and report the findings of stroboscopy, the authors note that 49 different parameters were used in the included studies, each associated with up to 15 different terms and 24 different scales. This fact indicates a severe lack of agreement on which parameters should be assessed to measure voice treatment outcomes and on the terms used to define the parameters without ambiguity  [7].

Only some of the parameters evaluated have sufficient reliability and clinical relevance, such as the straightness of the edge of the vocal fold, the mucosal wave, and the extent of non-vibrating portions of the vocal fold [3, 8, 9].

Correlations between laryngostroboscopic measures and voice quality have rarely been reported. Uloza et al. used four parameters of video laryngostroboscopy, such as glottal closure, regularity of vibrations, mucosal wave, and symmetry of vibrations on a 100 mm visual analogue scale. They examined healthy and pathologic subjects and found substantial to almost perfect inter-rater reliability. The statistically significant moderate correlations of glottal closure and objectively measured relative glottal area confirmed the reliability of the glottal closure parameter [4]. Mes et al. [10] evaluated in a systematic review on laryngeal reinnervation based on 42 studies the videolaryngostroboscopic parameters glottal closure, vocal fold position, mucosal wave, phase symmetry, amplitude, and regularity, typically on an ordinal scale from 0 to 3. They found for videolaryngostroboscopy that the glottal gap is the only numeric parameter estimated by visual analysis. None of the reviewed studies used outcome parameters related to structural changes after laryngeal reinnervation. To our knowledge, the laryngostroboscopic findings themselves have not yet been used numerically as an outcome measure.

The purpose of this study was to apply a significantly reduced laryngostroboscopy evaluation protocol with ordinal scale parameters to an intervention study for unilateral vocal fold paralysis. Validation of such a protocol involves a high correlation with the outcome parameters in clinical interventional studies for the treatment of voice disorders, such as auditory perception of voice quality, which remains the gold standard in the diagnosis of voice disorders. The question to be answered is: Does an index derived from laryngostroboscopic parameters correlate with voice quality, and can this index be used as an outcome measure?

## Methods

### Characteristics of the subjects

Twenty-six subjects with permanent unilateral vocal fold paralysis (UVFP) who had undergone surgery and for whom a complete preoperative and postoperative data set was available were identified in our clinical database. Seventeen subjects underwent Type I thyroplasty with autologous cartilage combined with arytenoid adduction (TP+AA) and nine a non-selective laryngeal reinnervation with nerve-to-nerve microsuture of parts of the ansa cervicalis to the recurrent laryngeal nerve (ANSA-RLN). For detailed patient data, see Table 1.

**Table 1:** Subjects data

Characteristic	N=26
Age, Median (Q1, Q3, Min, Max) in years	50 (34, 54, 17, 57)
Sex, n	
female	17
male	9

### Data assessment

#### Recording of the laryngostroboscopic data

All subjects were scoped and recorded preoperatively and postoperatively with a rigid 70° laryngostroboscope (XION medical, Berlin, Germany) with high-definition resolution at a comfortable pitch and loudness. The video clips were clipped to obtain sequences of one or two sustained vocalisations, including the previous inspiration and the vocal onset and offset. Six experienced physicians specialized in phoniatrics from our clinic and six from external departments rated the recordings. The instruction given was: “Please evaluate the endoscopic images according to the following scheme (see Table 2)!” All of them made their decisions independently and could not influence each other. The recordings were randomized, and the raters were blinded to the pre- or postoperative status.

#### Evaluating laryngostroboscopic data with the Laryngostroboscopy Index – LSI-UVFP

A newly designed and greatly reduced evaluation form shown in Table 2 was applied. It consisted of only four items that were expected to be most significant in the given clinical context of unilateral vocal fold paralysis:

- The **position** of the paralysed vocal fold **during respiration** can be median, paramedian, intermediate, or lateral [11]. Regarding position, the more lateral the vocal fold, the less closure is possible during phonation by moving the healthy vocal fold to the midline.
- **Atrophy** of the vocal fold due to denervation is the muscular loss of the thyroarytenoid muscle. The extent of atrophy depends on the degree of spontaneous reinnervation, which can vary.
- **Glottal closure** describes the configuration of the glottis at the moment of maximum approximation of the vocal folds **during the phonatory cycle**. A glottal gap during phonation allows greater air leakage. A larger gap also prevents contact between the vibrating vocal folds, so they cannot vibrate efficiently. Hence, the vocal quality decreases.
- The** mucosal wave** is the gliding of the vocal fold epithelium on the *M. vocalis*. When the muscle contracts and stiffens, the epithelium floating on the lamina propria is displaced laterally as a wave. This wave requires a solid muscle base, depending on the degree of reinnervation.

To assess potential overlaps between the four items, a correlation analysis using Kendall’s Tau was conducted. The resulting correlations yielded values of less than 0.6, indicating a high degree of independence. To determine the weighting factors for all four items and to obtain the sum of these weighted items, which was further named the Laryngostroboscopy Index for unilateral vocal fold paralysis (LSI-UVFP), a Principal Component Analysis was performed. LSI-UVFP is an ordinal variable, and the effect of a one-point increase is not mathematically constant across the entire scale. But for pragmatic purposes, for all subsequent analyses, LSI-UVFP was considered as an integer value.

In total, 624 single values were specified, corresponding to two ratings (preoperative and postoperative) per subject from 12 raters, respectively.

**Table 2:** Four items of the stroboscopic index

	normal	*→ *	*→ *	pathologic
Position of pVF	median	paramedian	intermediate	lateral
Atrophy of pVF	none	mild	moderate	severe
Glottal closure	complete	small gap	medium gap	large gap
Mucosal wave	normal	slightly reduced	strongly reduced	none
Score	0	1	2	3

Abbreviation: pVF: paralysed vocal fold

#### Recording of the voice data

For all subjects, preoperative and postoperative voice recordings were obtained. For the recordings, each patient was seated in a quiet room (background noise <40 dB) with a USB-mounted microphone (XION medical, Berlin, Germany) at a distance of 30 cm from the mouth following the recommendations of the European Laryngological Society [12]. All subjects were asked to read a standard text, the German version of ”The North Wind and the Sun” by Æsop.

#### Group Rating by Auditory Perceptual Evaluation (APE)

For the rating of voice quality, the second sentence of each recording was used. The voice samples were normalised for loudness using Audacity software V3.0.0 https://www.audacityteam.org/ and presented in randomised order and also blinded to the pre- or postoperative status using Praat phonetics software [13].

The rating group consisted of 15 speech and language pathologists and medical doctors from our department. They were asked to rate the question “How does that voice sound for you?” on a visual analogue scale from 0 (“very bad”) to 100 (“very good”), following the methodology for the “overall severity”-criterion of the “Consensus Auditory-Perceptual Evaluation of Voice (CAPE-V” protocol [14] and adopting the procedure used by Paniello et al. [15]. Each voice sample was rated twice and for analysis. The mean of both ratings was used, resulting in a total of 780 single values based on 1560 single ratings. The resulting voice quality rating scores of this perceptive rating are in the following referred to as APE for auditory perceptual evaluation.

### Statistics

To evaluate the internal validity and reliability of both ratings, several two-way intraclass correlation coefficients (ICC) were determined. Firstly, ICC(A,1) was used to measure the absolute agreement for single-rater measurements under a random-effects model. Secondly, ICC(C,1) was utilized to determine consistency in a test-retest setup involving different raters for the pre- and postoperative ratings.

To quantify the correlation between the medians (averaged across the raters) of the four items and LSI-UVFP, each with respect to APE, Pearson’s correlation coefficients were computed. Medians, averaged across the raters, of both LSI-UVFP and APE were analysed for each subject. Furthermore, Pearson’s correlation coefficient was computed to quantify correlation.

To quantify the effect of the predictor variable LSI-UVFP, time point (preoperative and postoperative) and sex, including their interaction, on the interval scaled APE, a linear mixed-effect model was built. The model also included the rater and the subject as random intercept effects. The quality of the model was determined by marginal and conditional R^2^. To test the significance of terms, a one-way ANOVA was used, choosing a confidence level of 95 %. All statistical calculations were performed in R statistics (v4.6.1, [16]).

## Results

The Principal Component Analysis of the four items showed that the first principal component covered 66.1 % (Table S4) of the variance with loadings of 0.42 to 0.63 (Table S5) for the four items. For pragmatic reasons, therefore, all weighting factors were set to 1 for each item. Finally, the LSI-UVFP resulted from the sum of the entries for the four items. Therefore, greater pathological changes of the larynx are reflected in higher LSI-UVFP values.

The ICC(A,1) showed good reliability for both scores: the APE (ICC(A,1) = 0.81) and the LSI-UVFP rating (ICC(A,1) = 0.80). ICC(C,1) = 0.60 for LSI-UVFP and 0.68 for APE proved moderate reliability.

**Fig. 1:**
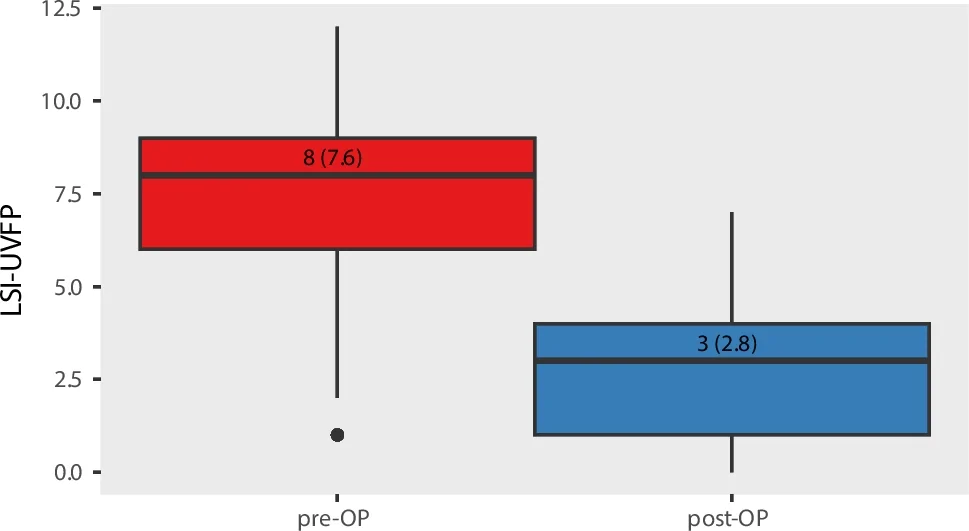
Boxplots of the Laryngostroboscopy Index - Unilateral Vocal Fold Paralysis (LSI-UVFP) values grouped by Time-point pre- (pre-op, red boxplot) and post-operatively (post-op, blue boxplot). The numbers within each box denote the median and the mean value (in parenthesis) for N=12 raters

**Fig. 2:**
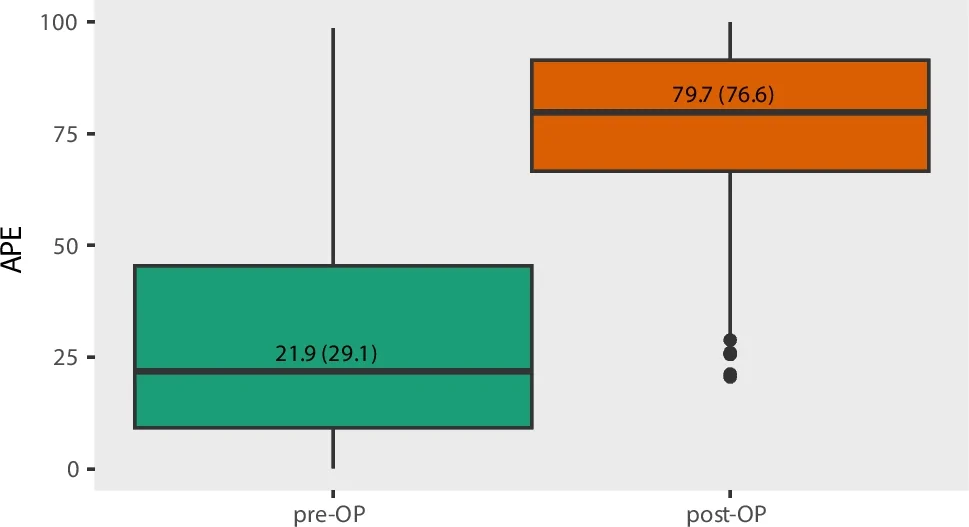
Boxplots of the Auditory Perceptual Evaluation (APE) values grouped by Time-point pre- (pre-op, green boxplot) and post-operatively (post-op, brown boxplot). The numbers within each box denote the median and the mean value (in parenthesis) based on N=15 raters

As seen in Fig. 1, the LSI-UVFP ratings of laryngostroboscopy by the 12 raters show median values of 8.0 (mean 7.6) preoperatively and median values of 3.0 (mean 2.8) postoperatively. The corresponding voice quality ratings show a median value of 21.9 (mean 29.1) for the 15 raters preoperatively and a median value of 79.7 (mean 76.6) postoperatively (see Fig. 2). For both measures, the boxes representing the inter-quartile range do not overlap, indicating a clear separation between the pre- and post-conditions.

The same applies to the individual values for all patients, averaged between the raters, for both ratings. And there were no apparent differences between the two sexes. The sex-independent Pearson correlation coefficient for LSI-UVFP was determined to be -0.76, which corresponds to a strong negative correlation. A strong negative correlation was also determined for all four items, i.e., -0.66 for position, -0.73 for glottal closure, -0.66 for mucosal wave, and -0.65 for atrophy.

**Fig. 3:**
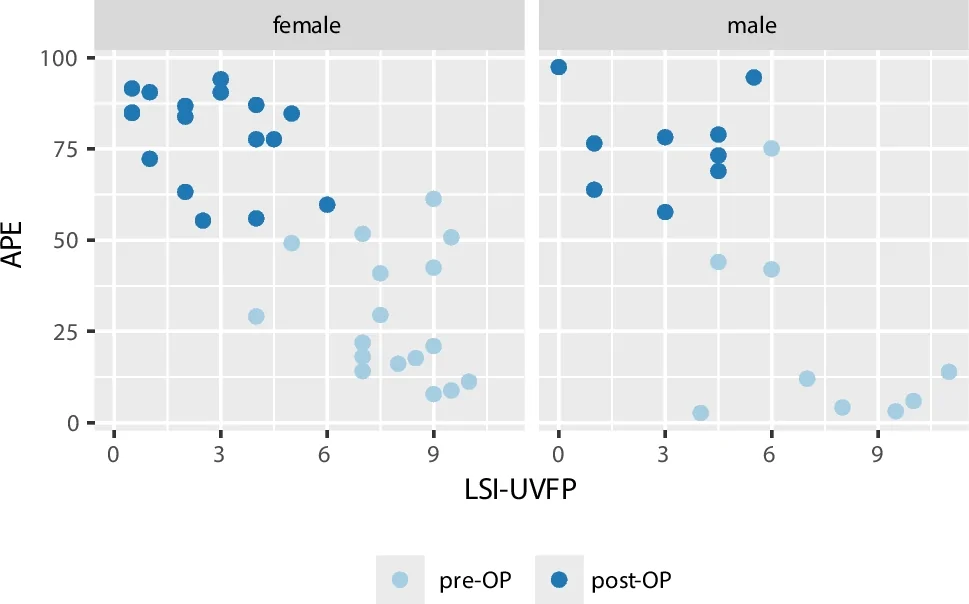
Medians (grouped by raters) of respective ratings for Auditory Perceptual Evaluation (APE) vs. Laryngostroboscopy Index-Unilateral Vocal Fold Paralysis (LSI-UVFP) for single subjects and splitted by sex. Points in light blue are the medians for pre-operative (pre-OP) ratings, whereas dark blue medians are the medians for post-operative (post-OP) ratings

Based on the linear mixed-effect model, no significant interaction was detected between LSI-UVFP, time point, and sex. Neither did sex significantly determine the model (Table S9). Therefore, the model could be reduced so that LSI-UVFP (p*=*.0002, Table S10) and time point (p<.0001, Table S10) were the only predictor variables. The marginal R$^2$ was calculated as 0.57, while the conditional R$^2$ was calculated as 0.77, indicating an improved model considering random effects.

APE increased by 40.2 units (standard error, S.E.=3.84, p<.0001, Table S11) when the time point changes from preoperative to postoperative and APE decreased by 2.45 units (S.E.=0.64, p*=*.0002, Table S11) when LSI-UVFP increases by one unit.

## Discussion

Until now, laryngostroboscopic data have been used in clinical studies primarily as a descriptive tool. This is surprising considering the importance of this examination technique in daily clinical routine. The difficulties lie in the complexity of evaluating laryngostroboscopic data and its various influential factors. As Poburka [2] pointed out, the scores are only meaningful if the patients achieve normal pitch and loudness during the examination. Impaired voices often do not meet these technical minimum requirements, and the range of vocal limitations caused by laryngeal diseases is wide, as is the number of parameters. This background situation limits the reliability of laryngostroboscopic data.

To answer the question of which parameters should be assessed to measure voice treatment outcomes, their stroboscopic ratings must be reliable. In a recent study, the application of the Rosen Stroboscopy Research Instrument [3] proved insufficient for analysis due to numerous missing values [17]. This could be one reason why it has not yet been possible to establish a universal stroboscopy rating for laryngeal diseases that can be used for evaluation in clinical studies. Since LSI-UVFP focuses on the parameters that are essential for UVFP, the assessment yields consistent, comprehensive, and–crucially–clinically relevant data.

In the presented approach, we focus on a selected group of patients whose laryngostroboscopic data were expected to be meaningful and coherent. In cases with UVFP, the voices become more breathy and weaker. However, the glottal vibration pattern allows for a reliable evaluation by laryngostroboscopy. The four parameters included in the LSI-UVFP reflect the extent of denervation, as well as the extent of functional deficits in voice production in UVFP. The position of the paralysed VF becomes more lateral as the laryngeal muscles lose tone. The same holds for muscle atrophy. Both the position of the VF and the degree of atrophy are closely correlated with voice quality. Yet, existing video-stroboscopic evaluation protocols do not include the degree of atrophy and neglect the position of the (paralysed) vocal fold completely. The two other parameters of the index, glottal closure in the closed phase of the stroboscopic cycle when the vocal folds are closest to each other and mucosal wave, are common features for any stroboscopic evaluation. They are also related to voice quality and seem to depend on the position of the VF and the degree of atrophy. Thus, the combination of the selected LSI-UVFP parameters (see Table 2) provides a good representation of both, the structural and morphological changes, as well as the functional limitations of the unilaterally denervated larynx. In particular, the addition of the purely morphological categories of atrophy and position enables a comprehensive assessment, making it possible to incorporate standardly acquired image data into the evaluation of therapeutic effects. However, it is unlikely that the selected parameter combination will show an equally good correlation for other laryngological pathologies that lack corresponding muscle atrophy or a change in vocal fold position.

The evaluation of the LSI-UVFP is carried out on four levels, each with integer steps from 0 to 3. The four-level classification has disadvantages when it comes to accurately assessing smaller differences. This can be seen as a limitation. However, in the context of the study, it has proven to be sufficient to reliably distinguish between preoperative and postoperative status. Furthermore, the chosen concept of a reduced index is supported by our finding that LSI-UVFP correlates well with rated voice quality, represented by APE (see Fig. 3 and Figure S1). Additionally, the standard deviations determined correspond to those obtained from the auditory evaluation.

In a recent review, the three main structural outcomes of a laryngeal reinnervation for UVFP are muscle bulk, tension, and position of the arytenoid [10]. All three are represented by the newly designed LSI-UVFP. The Index provides a validated evaluation tool based purely on video-endoscopic data and correlates well with a perceptive group rating of voice quality. LSI-UVFP should contribute to a more detailed and improved evaluation of the results after surgery.

## Conclusion

The proposed laryngostroboscopy index for unilateral vocal fold paralysis (LSI-UVFP) shows a good correlation with an auditory-perceptual group rating of voice quality for a group of patients with unilateral paralysis of the vocal fold before and after a voice-improving surgery, as demonstrated here based on laryngeal reinnervation in the ansa technique and thyroplasty with arytenoid adduction. Its usage enables a meaningful interpretation of laryngostroboscopic data, which are part of most clinical study evaluation protocols and could thus be effectively included in comparative pre-post evaluations for this group of patients and can serve as an endpoint measure.

## Supplementary Information

Below is the link to the electronic supplementary material.

Supplementary file 1 (pdf 410 KB)

Supplementary file 2 (Rmd 22 KB)

Supplementary file 3 (xlsx 84 KB)

## Data Availability

The raw data are published alongside the paper as supplementary material.

## Code availability

The R-script and statistical analysis are published alongside the paper as supplementary material.
