# Supplementary material for: An easy-to-use laryngostroboscopy index for unilateral vocal fold paralysis reflects voice quality
Source: Eur Arch Otorhinolaryngol. 2026 Sep 16;283(11):7331–6. doi: 10.1007/s00405-026-10575-4 (PMC13748058; doi:10.1007/s00405-026-10575-4)
Supplement: Supplementary file 1 [file 405_2026_10575_MOESM1_ESM.pdf]

Data analysis (2026-07-14)

An Easy-to-Use Laryngostroboscopy Index for Unilateral Vocal Fold Paralysis Reflects Voice Quality

Contents

1 Data **2**

2 Evaluating weighting factors based on Principal-Component-Analysis (PCA) **3**

3 Discriminant validity of the items **3**

4 Reliability **3**

4.1 Inter-Rater-Reliability only 3

4.2 Test-Retest- and Inter-Rater-Reliability (fully crossed design) 3

5 LSI-UVFP: Grouped data **4**

6 APE: Grouped data **4**

7 LSI-UVFP vs. APE: Pearsons correlation coefficients of grouped data **4**

8 APE vs. LSI-UVFP: linear mixed-effect model (full data) **4**

9 Package info **6**

1 Data

Table S1: General overview (Patients)

**Characteristic**	**N = 26**
Age, Median (Q1, Q3, Min, Max)	50 (34, 54, 17, 57)
Sex, n (%)	
female	17 (65.4%)
male	9 (34.6%)

Table S2: Data statistics (LSI-UVFP)

**Characteristic**	**pre-OP** N = 312	**post-OP** N = 312
RaterID, n (%)		
11	26 (8.3%)	26 (8.3%)
13	26 (8.3%)	26 (8.3%)
14	26 (8.3%)	26 (8.3%)
15	0 (0.0%)	0 (0.0%)
16	0 (0.0%)	0 (0.0%)
17	0 (0.0%)	0 (0.0%)
18	0 (0.0%)	0 (0.0%)
19	26 (8.3%)	26 (8.3%)
20	0 (0.0%)	0 (0.0%)
21	0 (0.0%)	0 (0.0%)
22	0 (0.0%)	0 (0.0%)
23	0 (0.0%)	0 (0.0%)
24	0 (0.0%)	0 (0.0%)
25	26 (8.3%)	26 (8.3%)
26	0 (0.0%)	0 (0.0%)
100	26 (8.3%)	26 (8.3%)
101	26 (8.3%)	26 (8.3%)
102	26 (8.3%)	26 (8.3%)
103	26 (8.3%)	26 (8.3%)
104	26 (8.3%)	26 (8.3%)
105	26 (8.3%)	26 (8.3%)
106	26 (8.3%)	26 (8.3%)
Sex, n (%)		
female	204 (65.4%)	204 (65.4%)
male	108 (34.6%)	108 (34.6%)
LSIUVFP, Median (Q1, Q3, Min, Max)	8.0 (6.0, 9.0, 1.0, 12.0)	3.0 (1.0, 4.0, 0.0, 7.0)

Table S3: Data statistics (APE)

**Characteristic**	**pre-OP** N = 390	**post-OP** N = 390
RaterID, n (%)		
11	26 (6.7%)	26 (6.7%)
13	26 (6.7%)	26 (6.7%)
14	26 (6.7%)	26 (6.7%)
15	26 (6.7%)	26 (6.7%)
16	26 (6.7%)	26 (6.7%)
17	26 (6.7%)	26 (6.7%)
18	26 (6.7%)	26 (6.7%)
19	26 (6.7%)	26 (6.7%)
20	26 (6.7%)	26 (6.7%)
21	26 (6.7%)	26 (6.7%)
22	26 (6.7%)	26 (6.7%)
23	26 (6.7%)	26 (6.7%)
24	26 (6.7%)	26 (6.7%)
25	26 (6.7%)	26 (6.7%)
26	26 (6.7%)	26 (6.7%)
100	0 (0.0%)	0 (0.0%)
101	0 (0.0%)	0 (0.0%)
102	0 (0.0%)	0 (0.0%)
103	0 (0.0%)	0 (0.0%)
104	0 (0.0%)	0 (0.0%)
105	0 (0.0%)	0 (0.0%)
106	0 (0.0%)	0 (0.0%)

Sex, n (%)		
female	255 (65.4%)	255 (65.4%)
male	135 (34.6%)	135 (34.6%)
APE, Median (Q1, Q3, Min, Max)	22 (9, 46, 0, 98)	80 (67, 91, 21, 100)

2 Evaluating weighting factors based on Principal-Component-Analysis (PCA)

Table S4: Results, Principal-Component-Analysis (PCA)

PC	Standard deviation	Proportion of Variance	Cumulative Proportion
1	1.6477085	0.66087	0.66087
2	0.7746013	0.14605	0.80692
3	0.6988558	0.11889	0.92581
4	0.5520857	0.07419	1.00000

Table S5: Loads, first PC

item	loads
Atrophie	0.4609882
Stellung	0.4185763
Glottisschluß	0.6272671
RKV	0.4677817

3 Discriminant validity of the items

Table S6: Correlation between four items (Kendalls' Tau)

	Atrophie	Glottisschluß	RKV	Stellung
Atrophie	1.0000000	0.5402102	0.4013976	0.4192801
Glottisschluß	0.5402102	1.0000000	0.5863393	0.5213632
RKV	0.4013976	0.5863393	1.0000000	0.3353242
Stellung	0.4192801	0.5213632	0.3353242	1.0000000

4 Reliability

4.1 Inter-Rater-Reliability only

ICC(A,1) (LSI-UVFP): 0.8 (good reliability) ICC(A,1) (APE): 0.81 (good reliability)

4.2 Test-Retest- and Inter-Rater-Reliability (fully crossed design)

ICC(C,1) (LSI-UVFP): 0.6 (moderate reliability) ICC(C,1) (APE): 0.68 (moderate reliability)

5 LSI-UVFP: Grouped data

Table S7: Group statistics (LSI-UVFP)

**Characteristic**	**pre-OP** N = 26	**post-OP** N = 26
Sex, n (%)		
female	17 (65.4%)	17 (65.4%)
male	9 (34.6%)	9 (34.6%)
LSIUVFP, Median (Q1, Q3, Min, Max)	7.75 (7.00, 9.00, 4.00, 11.00)	3.00 (1.00, 4.50, 0.00, 6.00)

6 APE: Grouped data

Table S8: Group statistics (APE)

**Characteristic**	**pre-OP** N = 26	**post-OP** N = 26
Sex, n (%)		
female	17 (65.4%)	17 (65.4%)
male	9 (34.6%)	9 (34.6%)
APE, Median (Q1, Q3, Min, Max, Mean)	20 (11, 42, 3, 75, 27)	79 (69, 87, 55, 97, 78)

7 LSI-UVFP vs. APE: Pearsons correlation coefficients of grouped data

LSI-UVFP: -0.76

Stellung/position: -0.66

Glottisschluß/glottal closure:-0.73

RKV/mucosal wave: -0.66

Atrophie/atrophy: -0.65

8 APE vs. LSI-UVFP: linear mixed-effect model (full data)

Table S9: APE ∼ LSIUVFP * Timepoint * Sex + (1 | RaterID) + (1 | ID) (ANOVA(LMER); R2: 0.77/0.57 conditional/marginal)

	Sum Sq	Mean Sq	NumDF	DenDF	F value	Pr(>F)
LSIUVFP	4446.98193	4446.98193	1	248.91708	15.4121748	0.0001119
Timepoint	7948.46153	7948.46153	1	242.91392	27.5474649	0.0000003
Sex	113.32367	113.32367	1	84.69736	0.3927527	0.5325416
LSIUVFP:Timepoint	507.12494	507.12494	1	240.84774	1.7575736	0.1861835
LSIUVFP:Sex	98.99791	98.99791	1	247.67885	0.3431031	0.5585769
Timepoint:Sex	581.79215	581.79215	1	241.53669	2.0163523	0.1569020
LSIUVFP:Timepoint:Sex	1051.14113	1051.14113	1	240.40051	3.6430035	0.0574962

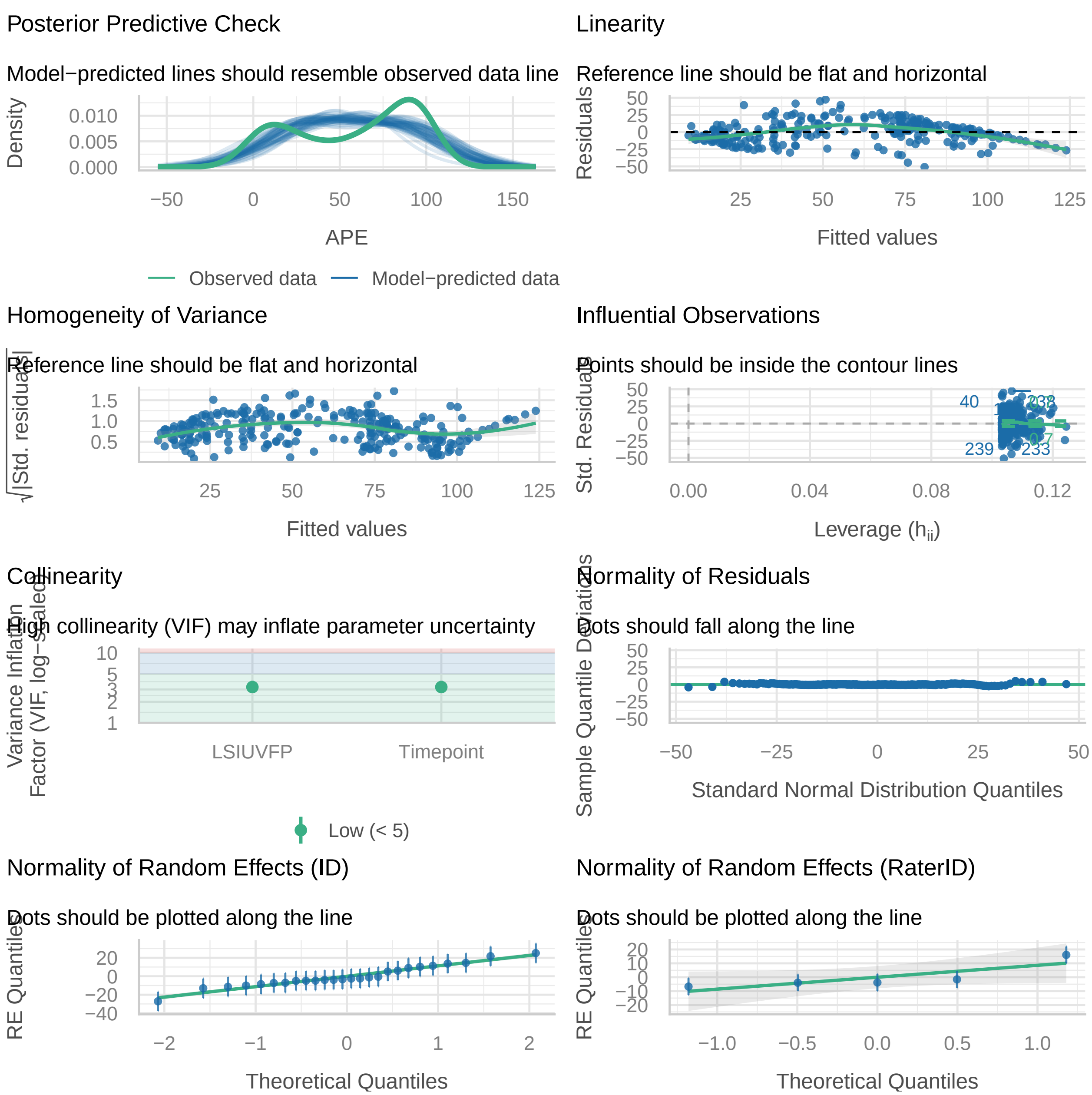

Figure S1: Model quality check (lmer)

Table S10: APE ~ LSIUVFP + Timepoint + (1 | RaterID) + (1 | ID) (ANOVA(LMER); R2: 0.77/0.57 conditional/marginal)

	Sum Sq	Mean Sq	NumDF	DenDF	F value	Pr(>F)
LSIUVFP	4253.409	4253.409	1	254.7842	14.43628	0.0001814
Timepoint	32311.580	32311.580	1	254.6104	109.66709	0.0000000

Table S11: APE ~ LSIUVFP + Timepoint + (1 | RaterID) + (1 | ID) (SUMMARY(LMER))

	Value β	S.E.	df	t.value	p.value
(Intercept)	49.917303	7.0266118	24.69016	7.104036	0.0000002
LSIUVFP	-2.450743	0.6450155	254.78423	-3.799511	0.0001814
Timepointpost-OP	40.166238	3.8355088	254.61044	10.472206	0.0000000

9 Package info

```r
sessionInfo()
```

```
## R version 4.6.1 (2026-06-24)
## Platform: x86_64-pc-linux-gnu
## Running under: Ubuntu 24.04.4 LTS
##
## Matrix products: default
## BLAS:   /usr/lib/x86_64-linux-gnu/openblas-pthread/libblas.so.3
## LAPACK: /usr/lib/x86_64-linux-gnu/openblas-pthread/libopenblasp-r0.3.26.so;  LAPACK version 3.12.0
##
## locale:
##  [1] LC_CTYPE=de_DE.UTF-8       LC_NUMERIC=C
##  [3] LC_TIME=de_DE.UTF-8        LC_COLLATE=de_DE.UTF-8
##  [5] LC_MONETARY=de_DE.UTF-8    LC_MESSAGES=de_DE.UTF-8
##  [7] LC_PAPER=de_DE.UTF-8       LC_NAME=C
##  [9] LC_ADDRESS=C               LC_TELEPHONE=C
## [11] LC_MEASUREMENT=de_DE.UTF-8 LC_IDENTIFICATION=C
##
## time zone: Europe/Berlin
## tzcode source: system (glibc)
##
## attached base packages:
## [1] stats     graphics  grDevices utils     datasets  methods   base
##
## other attached packages:
##  [1] ordinal_2025.12-29 janitor_2.2.1      broom_1.0.13       gt_1.3.0
##  [5] gtsummary_2.5.1    kableExtra_1.4.0   RColorBrewer_1.1-3 lubridate_1.9.5
##  [9] forcats_1.0.1      stringr_1.6.0      dplyr_1.2.1        purrr_1.2.2
## [13] readr_2.2.0        tidyr_1.3.2        tibble_3.3.1       tidyverse_2.0.0
## [17] openxlsx_4.2.8.1   ggpmisc_0.7.0      ggpp_0.6.0         ggplot2_4.0.3
##
## loaded via a namespace (and not attached):
##  [1] tidyselect_1.2.1    viridisLite_0.4.3   farver_2.1.2
##  [4] S7_0.2.2            fastmap_1.2.0       TH.data_1.1-5
```

```
##  [7] bayestestR_0.18.1   digest_0.6.39       estimability_2.0.0
## [10] timechange_0.4.0    lifecycle_1.0.5     survival_3.8-6
## [13] magrittr_2.0.5      compiler_4.6.1      rlang_1.2.0
## [16] tools_4.6.1         yaml_2.3.12         knitr_1.51
## [19] labeling_0.4.3      xml2_1.6.0          irr_0.85
## [22] multcomp_1.4-30     withr_3.0.3         numDeriv_2016.8-1.1
## [25] datawizard_1.3.1    grid_4.6.1          emmeans_2.0.3
## [28] scales_1.4.0        MASS_7.3-65         tinytex_0.60
## [31] insight_1.5.2       dichromat_2.0-0.1   mvtnorm_1.4-1
## [34] cli_3.6.6           rmarkdown_2.31      ragg_1.5.2
## [37] reformulas_0.4.4    generics_0.1.4      otel_0.2.0
## [40] rstudioapi_0.19.0   performance_0.17.0  tzdb_0.5.0
## [43] parameters_0.29.2   minqa_1.2.8         polynom_1.4-1
## [46] splines_4.6.1       vctrs_0.7.3         sandwich_3.1-1
## [49] boot_1.3-32         Matrix_1.7-5        SparseM_1.84-2
## [52] patchwork_1.3.2     hms_1.1.4           ggrepel_0.9.8
## [55] systemfonts_1.3.2   see_0.14.1          glue_1.8.1
## [58] nloptr_2.2.1        codetools_0.2-20    stringi_1.8.7
## [61] gtable_0.3.6        lme4_2.0-1          lmerTest_3.2-1
## [64] pillar_1.11.1       htmltools_0.5.9     quantreg_6.1
## [67] R6_2.6.1            ucminf_1.2.3        textshaping_1.0.5
## [70] Rdpack_2.6.6        evaluate_1.0.5      lpSolve_5.6.23
## [73] lattice_0.22-9      rbibutils_2.4.1     backports_1.5.1
## [76] cards_0.8.0         snakecase_0.11.1    MatrixModels_0.5-4
## [79] Rcpp_1.1.1-1.1      zip_3.0.0           coda_0.19-4.1
## [82] svglite_2.2.2       nlme_3.1-169        mgcv_1.9-4
## [85] xfun_0.59           zoo_1.8-15          fs_2.1.0
## [88] pkgconfig_2.0.3
```
